# “CANDLE syndrome: A closer look at a rare autoinflammatory disorder”

**DOI:** 10.1016/j.jtauto.2025.100339

**Published:** 2025-12-06

**Authors:** Shivam Singh, Ashish Kumar Sharma

**Affiliations:** Department of Pharmacology, Nims Institute of Pharmacy, Nims University Rajasthan, Jaipur, 303121, Rajasthan, India

**Keywords:** CANDLE, Lipodystrophy, Dermatosis, Interventions, Biopsies

## Abstract

A newly identified autoinflammatory condition called CANDLE syndrome (chronic atypical neutrophilic dermatosis with lipodystrophy and increased temperature) is characterized by early onset, recurring fever, skin lesions, and multisystemic inflammatory symptoms. It has been demonstrated that the majority of patients had PSMB8 gene mutations. It leads to dysfunction in the proteasome/immunoproteasome system and subsequent overproduction of type 1 interferons. Patients usually exhibit lipodystrophy, fever, rashes on the skin, and malnutrition in the early stages of infancy. The results of skin biopsies, laboratory tests, and clinical symptoms all support the diagnosis of CANDLE syndrome. Although there isn't a specific treatment for CANDLE syndrome, JAK inhibitors like baricitinib have demonstrated some effectiveness in treating its symptoms. For CANDLE syndrome patients to receive the right therapeutic interventions, early diagnosis and molecular testing are essential. A positive interferon signature has also been found to be a diagnostic indicator for the condition. Although there are no particular treatments for CANDLE syndrome, research is still being done to determine how well immunosuppressive medications, biological agents, and glucocorticoids work in treating the condition. Current research generally aims to improve the quality of life for individuals with CANDLE syndrome through the development of targeted medications, the elucidation of genetic determinants, and the advancement of diagnostic methods.

## 1 Introduction

CANDLE syndrome was first identified in 2010 as a rare inherited autoinflammatory disease marked by persistent systemic inflammation, gradual loss of adipose tissue, and characteristic skin abnormalities. Most patients develop symptoms early in life. The disorder arises from mutations that impair the function of the proteasome or immunoproteasome, disrupting normal protein degradation. This dysfunction triggers uncontrolled inflammatory signaling, particularly sustained activation of type I interferon pathways [1,2]. An extensive report of four patients with early onset recurrent fevers, annular violaceous skin lesions made up of infiltrates of mature neutrophils and atypical mononuclear cells, lipodystrophy, and multisystemic inflammatory manifestations was published in 2010 [3]. An abbreviation for CANDLE syndrome was created as a result of these unique clinical characteristics [4]. Following investigations, a mutation in the human immunoproteasome gene was found, supporting the syndrome's genetic basis and classifying it within the PRAAS category [5,6]. The PSMB8 gene, which codes for the β5i subunit of the immunoproteasome, was shown to have homozygous or compound heterozygous mutations in patients with this syndrome [7,8]. Later research identified mutations in additional genes encoding distinct proteasome subunits, including the immunoproteasome, in patients diagnosed with CANDLE syndrome [9,10]. Less than 40 cases of CANDLE syndrome have been reported globally, and the majority of patients are of Spanish, Portuguese, Hispanic, or Japanese descent. There have also been reports of patients from the UK, Israel, Turkey, China, and the USA. According to function evaluation, these mutations affect transcription, protein production, folding, proteasome assembly, and, eventually, proteasome activity in different ways. Additionally, aberrant proteasome synthesis and activity were recreated in primary fibroblasts from healthy individuals using siRNA-mediated inhibition of the relevant subunits [11].

Regardless of genotype, the IFN gene-expression profile was strong in hematopoietic and nonhematopoietic cells separated from patients. The removal of misfolded proteins and the degradation of intracellular proteins coming from external or self-structures are important tasks performed by the ubiquitin proteasome system (UPS) [12]. The 20S core of the proteasome is composed of two outer α-rings and two inner β-rings arranged in the following pattern: α1–7β1–7β1–7α1–7. Proteolytically active sites that resemble caspase-like activity are present in the β1 subunit, trypsin-like activity is present in the β2 subunit, and chymotrypsin-like activity is present in the β5 subunit. The only known CANDLE/PRAAS-causing mutation is found in PSMB8, which encodes the alternative inducible catalytic proteasome subunit β5i (also called LMP7) [13,14]**.**

In order to create immunoproteasomes and increase proteolytic capacity, two additional inducible active sites, β1i (also expressed by PSMB9 and also known as LMP2) and β2i (also encoded by PSMB10 and also known as Mecl1), can be incorporated into growing proteasome complexes. Despite the fact that hematopoietic cells generally display immunoproteasome synthesis, proinflammatory cytokines in the majority of other organs cause it to occur. The aim of this manuscript is to present a clear understanding of the molecular and genetic mechanisms involved in CANDLE syndrome, with particular emphasis on how defects in the proteasome and excessive type I interferon activity contribute to disease development. This review also intends to outline the range of mutations in PSMB8 and other proteasome-related genes, describe how these alterations impair proteasome formation and function, and relate these abnormalities to the key clinical signs such as progressive lipodystrophy, recurrent fevers, and characteristic skin lesions. Additionally, the manuscript seeks to discuss recent progress in diagnostic tools including interferon-signature profiling and molecular genetic testing and to examine current and emerging therapeutic options, especially the growing use of JAK inhibitors and other targeted immunomodulators. Overall, the objective is to provide a cohesive overview that supports early recognition of the disorder, informs clinical management, and highlights areas where further investigation is needed to improve outcomes for individuals with CANDLE syndrome.

### 1.1 Genetic basis

Initially, mutations in PSMB8 were identified as the primary cause of Nakajo Nishimura syndrome, JMP syndrome, and CANDLE syndrome. Subsequent studies, however, revealed that patients with CANDLE like phenotypes may also harbour mutations in other genes encoding proteasome or immunoproteasome subunits, as well as in the proteasome assembly factor POMP. These discoveries expanded the genotypic spectrum of CANDLE syndrome. To date, the following genetic defects have been documented in affected individuals.

#### 1.1.1 Mutations in PSMB4

PSMB4, located on chromosome 1q21, encodes the β7 subunit of the proteasome, which plays a crucial role in proteasome stability and assembly. The c.-9G > A mutation results in reduced expression of the β7 subunit compared with the wild-type protein, leading to decreased incorporation into proteasome complexes. This defect is thought to impair the proper cleavage of pro-peptides by the β5i subunit.

A deletion affecting three amino acids (p.D212–V214) alters the N-terminus of an α-helix and disrupts the intramolecular hydrogen-bonding network required to stabilize the C-terminal extension of β7 a region essential for proteasome assembly. Additional mutations include.

• p.Y222X (nonsense mutation): causes loss of the β7 C-terminal extension.
• c.44insG (frameshift; p.P16Sfs∗45): prevents expression of the mutant allele.

Even when expressed, mutant β7 proteins fail to incorporate into the 20S and 26S proteasome complexes, resulting in impaired proteasome maturation [15].

#### 1.1.2 Mutations in POMP

The POMP gene, located on chromosome 13q12.3, encodes the proteasome maturation protein, an essential chaperone that facilitates the sequential incorporation of β-subunits into the assembling proteasome. Although most CANDLE cases involve mutations in proteasome subunit genes, one reported patient exhibited no such mutations but instead carried a heterozygous, dominant frameshift insertion in POMP (c.344_345insTTTGA; p.E115Dfs∗20).

This mutation produces a truncated and likely unstable POMP protein. Defective POMP leads to accumulation of immature proteasome precursors, markedly reduced assembly of mature proteasomes, and decreased overall proteasome catalytic activity [16].

#### 1.1.3 Mutations in PSMB8

PSMB8, located on chromosome 6p21.32, encodes the β5i subunit of the immunoproteasome, which possesses chymotrypsin-like proteolytic activity. During immunoproteasome maturation, the β5i precursor must undergo proteolytic removal of its prosequence; failure of this step disrupts assembly and function.

In CANDLE syndrome, PSMB8 mutations impair either.

• The chymotrypsin-like catalytic function, or
• Proper immunoproteasome assembly and maturation.

**Fig. 1:**
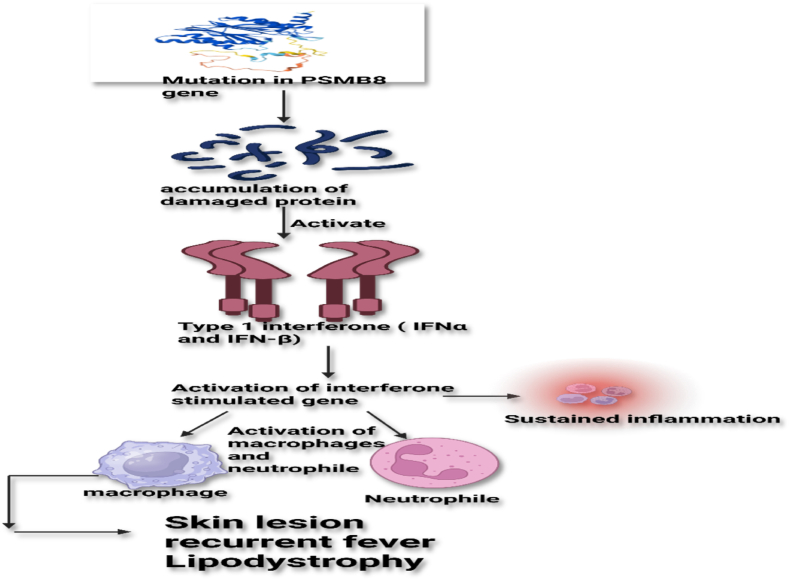
Pathogenic pathway of PSMB8 mutation leading to immunoproteasome dysfunction and chronic interferon activation in CANDLE syndrome.

Such dysfunction results in defective protein degradation, accumulation of ubiquitinated or misfolded proteins, and subsequent activation of type I interferon pathways. These molecular disturbances correlate with hallmark clinical features including fever, lipodystrophy, and neutrophilic dermatosis [17], as illustrated in Fig. 1.

#### 1.1.4 Mutations in PSMB9

PSMB9, also located on chromosome 6p21.32, encodes the β1i subunit of the immunoproteasome, responsible for caspase-like proteolytic activity. The only reported pathogenic variant to date is a missense mutation p.G165D, situated in a loop between two α-helices that contributes to the formation of the β1i active site. This structural disruption affects the threonine-mediated catalytic mechanism, thereby compromising β1i activity and immunoproteasome function [18].

## 2 Clinical manifestation

**Fig. 2:**
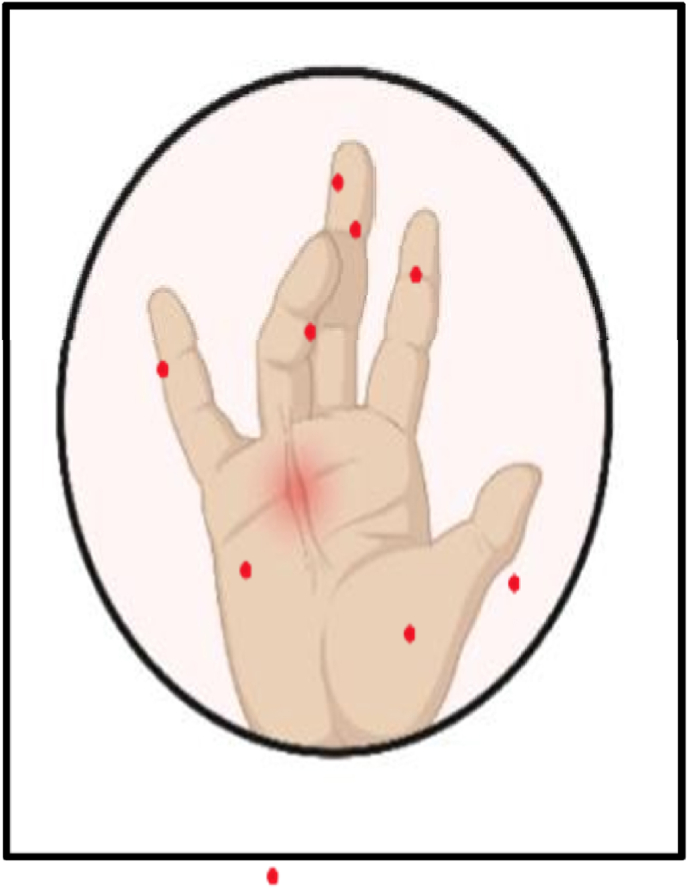
Fingertip ulceration and digital swelling in association with joint contractures.

**Fig. 3:**
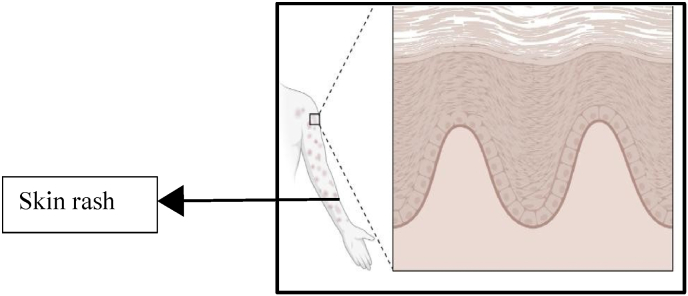
Structural illustration of healthy skin layers.

The unusual autoinflammatory condition known as CANDLE syndrome usually first manifests in early childhood and is characterized by persistent systemic inflammation and unique dermatological characteristics. Along with persistent skin rashes, sensitive nodules, and panniculitis-like lesions, patients typically suffer from repeated, protracted fever that are resistant to therapy. This often results in lipodystrophy or irregular distribution of fat. Developmental delays, gradual joint contractures and uveitis are possible additional symptoms. The chronic inflammatory state that defines the condition is reflected in laboratory results, which frequently display high inflammatory markers such as erythrocytes sedimentations rate and CRP. Some of clinical finding is mentioned below in Table 1 and Fig. 2, Fig. 3.

**Table 1:** Characteristic clinical and systemic manifestations of PRAAS/CANDLE syndrome.

Clinical manifestation	Description	Reference
Skin engrossment	Persistent skin lesion	[19]
Lipodystrophy	subcutaneous fat loss, specifically in extremities	[20]
Cardiomyopathy	Widening and fading of the heart muscle, leads to heart failure	[21]
Failure to Thrive	Deprived growth and weight gain in infancy.	[22]
Fever	irregular high fevers often existing from infancy	[23]
Recurrent Infections	Augmented exposure to infections due to immunological anomalies	[24]
Musculoskeletal Pain	Chronic pain in muscles leads to physical uneasiness and incapacity	[25]
Developmental delay	Delay in achieving developmental delay	[26]
Myositis	Inflammation of muscle tissue leads to paleness and aching	[27]
Elevated Inflammatory Markers	augmented levels of C-reactive protein CRP and ESR, cause chronic inflammation	[28]
Joint contractures	Stiffness and limited motion in joints, often occur at early in life	[29]
Uncharacteristic Facial Appearance	Distinctive facial structures involving periorbital swelling, triangular face, and bulging forehead	[30]
Anaemia and Thrombocytopenia	Low RBC and platelets, rise to fatigue and increase bleeding	[31]
Hepatosplenomegaly	Widening of the liver and spleen, often observed during physical investigation	[31]

## 3 Diagnosis and histopathological findings

The typical clinical findings in infancy include violaceous eyelid edema, low weight and height, annular violaceous plaques, almost daily temperatures above 38.5 °C, and progressive lipodystrophy. Together with the unique findings from the skin biopsy, all of these can help confirm the diagnosis. The histology of lesional skin shows mature neutrophils and atypical mononuclear infiltrates of myeloid ancestry [32]. Significant perivascular and interstitial infiltrates affecting the reticular and papillary dermis were found in the first four patients' repeated skin biopsies. Like leukemia, these aberrant cells seemed to represent an interstitial infiltration of myeloid lineage cells. They were mostly made up of mononuclear cells, which exhibited scant eosinophilic cytoplasm, a single inconspicuous nucleolus, and big, vesicular, elongated, or kidney-shaped nuclei. The myeloid lineage was confirmed by immunohistochemistry labelling with myeloperoxidase, lysozyme, Mac387, CD68 (KP1), and CD45. The sample contained a mixture of aberrant myeloid cells and mature neutrophils [33]. Laboratory abnormalities include high levels of alanine aminotransferase and aspartate aminotransferase, hypochromic anaemia, and raised acute-phase reactants, in addition to biopsy permission. A recently identified dysregulation of the IFN signalling pathway is also supported by genetic data demonstrating that mutations in PSMB8 cause CANDLE syndrome; all nine study patients had elevated, though variable, levels of IFNg inducible protein 10 (IP-10). Additional variable clinical features in the diagnosis of CANDLE include alopecia areata, hypertrichosis, and acanthosis nigricans [34,35].

### 3.1 Laboratory assessment of candle

As is the case with other AIDs, laboratory conclusions are not very prominent. The most common features are elevation of acute phase reactants ESR, CRP, and thrombocytosis and a chronic, hypochromic anaemia. Liver enzymes are moderately elevated, but this may be caused by lipodystrophy itself; also, enhanced triglyceride levels can occur in relation to metabolic disturbance by lipodystrophy. Elevated muscle enzymes CPK and aldolase less frequently indicate a persistent muscular contribution. Although autoimmunity and autoantibody tests are typically negative, some patients have elevated antinuclear antibody levels. Immunoglobulin serum levels are frequently normal. Biopsies of the lymph nodes and bone marrow have shown only reactive alterations and have been unremarkable [[36], [37], [38]].

### 3.2 Diagnosis of candle syndrome

Early development of fevers, skin lesions, and lipodystrophy are suggestive of CANDLE. A skin biopsy combined with immunohistochemistry analysis may have sufficient characteristics to provide a precise diagnosis. A confirmatory diagnosis is established by genetic analysis of the implicated genes. Certain characteristics of CANDLE syndrome may also be present in other AIDs, such as NOMID syndrome, TRAPS, or hyper-IgD syndrome [39]. While leprechaunism, acquired partial lipodystrophy of Barraquer-Simons, partial familial lipodystrophy, and widespread congenital lipodystrophy are all potential causes of fat loss, lipodystrophy is one of the most distinctive features of CANDLE syndrome. Aicardi-Goutières syndrome and other type 1 interferonopathies (such SAVI syndrome, familial chilblain lupus, or C1q deficiency) can also exhibit symptoms similar to those of CANDLE disease. Infants with Sweet syndrome may exhibit violaceous ring lesions that resemble CANDLE syndrome, and histology may not always be accurate. Clinical signs of otulipenia, a recently identified autoinflammatory condition caused by loss-of- function mutations in OTULIN, the gene encoding a deubiquitinase that cleaves Met1-linked chains, include skin lesions and fat loss [40,41]. Panniculitis is common in IFN- mediated syndromes. Proteasome-associated autoinflammatory syndromes (PRAAS) such as Nakajo-Nishimura syndrome and chronic atypical neutrophilic dermatosis with lipodystrophy and high temperature (CANDLE) syndrome are characterized by panniculitis. However, erythema nodosum is associated with the most prevalent clinical type of panniculitis and is frequently seen in patients with polygenic AIDSs, including sarcoidosis, Behçet's syndrome, and inflammatory bowel disease. Rare diseases such as otulipenia, lipoatrophic panniculitis in children, and cytophagic histiocytic panniculitis can also manifest as subcutaneous fat inflammation [42]. Patients with type I interferonopathies, such as CANDLE, SAVI, and AGS, often have a consistently elevated peripheral blood IFN signature. Conversely, individuals with SAVI and CANDLE/PRAAS typically exhibit elevated levels of traditional inflammatory markers such as erythrocyte sedimentation rate (ESR) and C- reactive protein (CRP), although AGS patients rarely do [43].

### 3.3 Management

The interdisciplinary approach to managing CANDLE syndrome focuses on several elements of the illness. There is currently no known cure for CANDLE syndrome, hence treatment choices are restricted. Nevertheless, some patients have responded favourably to biological therapies, immunosuppressive drugs, and glucocorticoids. Patients with CANDLE exhibit a robust type I interferon gene expression profile and respond well to JAK inhibitor therapy [4]. JAK inhibitors are a novel class of small molecule medications that have been utilized to treat numerous autoimmune and autoinflammatory conditions as well as uncommon oncologic disorders. Different degrees of success have been seen when using janus kinase inhibition for inflammatory bowel illnesses, lupus, psoriatic arthritis, and myeloproliferative diseases [44]. When compared to biological treatments, they exhibit promising outcomes in the treatment of adult rheumatoid arthritis, where they are particularly effective. Because JAK inhibitors are taken orally, patients find them to be extremely convenient. The article's description of the baricitinib treatment effect is consistent with previously released reports in CANDLE patients [45]. In several open-label trials, patients with type I interferonopathies receiving baricitinib treatment showed a significant decrease in their daily diary score and glucocorticoid use. None of the patients in the Sanchez et al. research had reached remission prior to starting baricitinib therapy, and 50 % of CANDLE/PRAAS patients experienced a long-lasting remission without any clinical symptoms, control of inflammatory markers while on baricitinib, and cessation of glucocorticoids [46,47]. In addition, patients with CANDLE have development in myositis and cytopenia (haemoglobin, lymphocyte and platelets). Additionally, JAKIs decrease IFN-α-mediated STAT-1 phosphorylation in a dose-dependent manner in patients with interferonopathy, thus indicating an in vivo effect of the JAK inhibitor on type I IFN signalling. The JAKIs, Ruxolitinib and tofacitinib, are also testified as possible treatment choices [48]. Patients with type I interferonopathy who received JAKI treatment have been documented to have viral reactivation, including BK viral reactivation. Renal transplant patients frequently experience BK polyomavirus reactivation due to therapeutic immunosuppression, which can lead to nephropathy and renal allograft failure. BK nephropathy has no known cure; the only methods of care are early identification and lowering the dosage of immunosuppressive drugs to control the BK virus load. Prior to starting JAKI, at baseline, and thereafter regularly at every visit, it is advised to monitor for the BK virus load in the blood and urine as well as renal function [49]. Despite their toxicity, glucocorticoids are typically thought to be helpful in CANDLE/PRAAS patients with systemic inflammation. Glucocorticoids have major negative effects, such as growth arrest, truncal obesity, hypertension, glucose intolerance, and osteopenia, when administered for extended periods of time. Consequently, the goal of glucocorticoid dosage reduction for illness management should be the lowest feasible level. Treatment for acute CNS and non-CNS inflammatory symptoms, such as hepatitis and cytopenia, may benefit from brief courses of glucocorticoids. Growth (stunting), development, and bone health (such as osteoporosis) are all significantly impacted by persistent inflammation and long-term glucocorticoid therapy. Regular monitoring is necessary for these indicators, as well as for cardiac (e.g., hypertension) and ophthalmologic problems resulting from long-term use of glucocorticoids [50,51]. Overview of Medications, Dosage Regimens, and Adverse Effects in CANDLE/PRAAS Management as shown in Table 2.

**Table 2:** Overview of medications, dosage regimens, and adverse effects in CANDLE/PRAAS management.

Treatment category	Medication	Dosage	Description	Adverseeffects
Corticosteroids	Prednisolone	Varies based on severity	Control acute flares; anti- inflammatory action	Weight gain, osteoporosis, hyperglycaemia
Immunomodulators	Methotrexate	7.5–2.5 mg weekly	Inhibit DNA synthesis	Nausea, liver toxicity, bone marrow suppression
	Azathioprine	1–3 mg/kg daily	Reduce immune activity, refractory cases	Bone marrow suppression, liver toxicity
Biologic agents	Etanercept (TNF inhibitor)	50 mg/kg weekly	Persistent inflammation	Injection site reaction, infections
	Anakinra (IL-1 inhibitor	100 mg daily	Severe inflammation	neutropenia
JAK inhibitor	Ruxolitinib	5–10 mg twice daily	For refractory cases target Janus kinase pathway	Anaemia, thrombocytopenia, infections
	tofacitinib	5 mg daily twice	Refractory cases	Infection, elevated liver enzymes
NSAIDS	ibuprofen	200–800 mg every 6–8 h	Pain relief, reduce inflammation	GIT irritation, renal impairment
	naproxen	250–500 mg twice daily	Mild to moderate inflammation and pain	Renal impairment
Supportive care	Analgesics	varies	Pain relief	Varies based on medications
	Nutritional supplement	As needed	Address nutritional deficiencies	Based on supplement
Antipyretics	acetaminophen	500 mg	fever	Liver toxicity
Experimental therapies	Emerging biologics	Clinical trial dosage	Target immune modulation	Varies based in experimental agents
	Antibiotics	As needed	Treat secondary infections	Antibiotic resistance, GIT issues

### 3.4 Research direction

#### 3.4.1 Understanding the molecular pathway involved in autoinflammation or CANDLE

**Sting pathway:** The immune system's highly conserved mechanism allows it to react to foreign DNA. Stimulator of Interferon Genes (STING), a major innate immunity protein housed in the endoplasmic reticulum, is an essential component of this system. It is a pattern recognition receptor that eventually mediates the production of type 1 interferon (IFN) after becoming activated in response to intracellular infection. Type I interferonopathies represent a group of inherited autoinflammatory conditions in which the interferon (IFN) signaling pathway becomes dysregulated. These disorders arise from mutations that disturb normal innate immune responses, particularly those involved in detecting and reacting to viral nucleic acids. As a result of this pathway imbalance, affected individuals may show a combination of autoinflammatory features, autoimmune manifestations, or varying degrees of immunodeficiency, reflecting the diverse biological consequences of the underlying genetic defects [52]. The gene TMEM173 encodes STING, which is expressed by T-cells, monocytes, natural killer cells, and dermal fibroblasts. It is made up of a cytoplasmic ligand binding and signalling domain, four transmembrane helices, and a domain-swapped dimer assembly. Double-stranded DNA (dsDNA) detection triggers the STING pathway by cyclic GTP-AMP-synthase (cGAS) cyclizing GTP and ATP to create 2′3′-cyclic GMP-AMP (cGAMP), the primary ligand that activates STING [53]. The ligand-binding domain closes in response to STING activation, which causes the ligand-binding domain to rotate 180° with respect to the transmembrane domain [54]. It has been discovered that the C-terminal tail of STING autoinhibits the protein by obstructing the inactive STING polymerization interface and thereby inhibiting autoactivation. The production of disulfide links is made possible by the conformational shift brought about by cGAMP, which also releases the CTT and exposes the polymer interface. As an alternative, bacterial cyclic-di-GMP can activate STING in concert with it, acting as a partial antagonist of the cGAMP signalling pathway. Studies in structure and biochemistry have offered sophisticated insight into the mechanism of STING differential by cGAMP and cDi-GMP [55]. Transcription factor IRF-3 is phosphorylated by STING-activated tank binding kinase 1 (TBK1), leading to its translocation to the nucleus. Most recently, three mutations of TMEM173 have been identified to directly induce STING-associated vasculopathy with onset in infants (SAVI). Gain-of-function mutations of TMEM173 inducing enhanced transcription of IFNs have been proven to cause autoimmune disorders such as CANDLE. According to a recent study, a novel therapeutic approach for STING inhibition involves the use of chemicals that generate a conformation distinct from that of cGAMP-bound STING [56,57]. Compared to earlier suggested medicines using STING inhibition, this approach would be less immunosuppressive and prevent constitutive STING activation in autoimmune disorders.

### 3.5 Developing targeted therapies

#### 3.5.1 Sting pathway inhibitors

Many illnesses are linked to abnormal innate immune pathway activation. Although the promise of targeted treatment methods has been enhanced by advances in our understanding of the molecular mechanisms behind innate immunity pathways, the creation of pharmaceuticals that selectively target desired molecules remains a difficult task. the identification and description of extremely strong and specific small-molecule inhibitors of the STING protein, a key signalling element in the intracellular DNA sensing pathway [58]. The discovered drugs prevent the activation-induced palmitoylation of STING by covalently targeting the anticipated transmembrane cysteine residue [59,60]. By employing these inhibitors, we demonstrate how STING's palmitoylation is necessary for its multimeric complex formation at the Golgi apparatus and, consequently, for the recruitment of downstream signalling components. In both human and mouse cells, the discovered chemicals and their derivatives inhibit the generation of inflammatory cytokines mediated by STING [61]. Additionally, we demonstrate that in mice, these small-molecule antagonists reduce the pathogenic aspects of autoinflammatory disease [62,63]. In summary, the process by which STING can be pharmacologically inhibited shows the promise of STING-targeting treatments for autoinflammatory diseases such as CANDLE/PRAAS [64].

#### 3.5.2 cGAS inhibitors: upstream targeting

An enzyme called cGAS is responsible for producing cGAMP in response to cytosolic DNA. By inhibiting the production of cGAMP, inhibitors targeting cGAS prevent STING activation [65,66].

#### 3.5.3 TBK1/IRF3 inhibitors: downstream blockade

Inhibitors directing TBK1 or IRF3 can block the signalling cascade downstream of STING stimulation, dropping the construction of type I interferons and other pro-inflammatory cytokines [[67], [68], [69]].

#### 3.5.4 CRISPR/Cas9

Bacteria and archaea possess a defense mechanism called the CRISPR/Cas9 system. The small, distinct "spacer" sequences that make up CRISPR sequences are inserted into segments of highly conserved repetitive DNA sequences, known as "CRISPR repeats [70]. CRISPR-associated cas genes are clusters of highly conserved protein-coding genes that are typically found next to these segments. These genes often have domains that resemble nucleases, helicases, polymerases, and nucleotide-binding proteins [71]. To create DSBs, the CRISPR/Cas9 system uses an endonuclease and a customized single-stranded guide RNA (sgRNA) [72]. The sgRNA is composed of a precursor crRNA with embedded spacer sequences that hybridize to a complementary trans-activating crRNA that is separately produced and has the complete length of CRISPR repeats [73]. The double stranded RNA-specific ribonuclease Cas9 is guided to any target location by the sgRNA. The target genome's conserved three-nucleotide species-specific proto adjacent motifs PAM are identified by the RNA-guided Cas9 enzyme as it scans the genome. CRISPR/Cas9 is quickly replacing other gene editing techniques because it is adaptable, effective, easy to develop and apply, and increasingly specific. Additionally, its range of usefulness is expanding [74].

#### 3.5.5 Genome editing

Changing the expression, structure, or function of effector proteins is the main objective of genome editing. For this purpose, gene editing has long been employed, which enables precise and effective DNA change at a particular locus or loci through the creation of a DSB [75]. After that, endogenous cellular machinery modifies 1 DSBs by facilitating either NHEJ or homologous recombination, also known as HDR [76,77]. By inserting the altered or wild-type DNA into its homologous target region in the genome, HDR fixes double-strand breaks DSBs. Even though HDR has been widely used in the past, its ability to modify genomic DNA specifically has been restricted due to its high rates of random template DNA insertion, off-target insertion, and limited functionality during the S and G2 cell cycle stages [78]. On the other hand, NHEJ, the most prevalent method of DSB repair in mammalian cells, is active at every stage of the cell cycle. Pathobiology research on inflammatory diseases has expanded thanks to advancements in genome editing technology [79]. Comparative Overview of Contemporary Genome and RNA Editing Technologies with benefits and drawbacks as reported in Table 3.

**Table 3:** Comparative overview of contemporary genome and RNA editing technologies with benefits and drawbacks.

Technology	Mechanism	Benefits	Drawbacks
CRISPR-Cas9	RNA-guided, DNA endonuclease	High precision and efficiency Cost effective Easy to design and implementation	Potential off-target effects ethical concern regarding germline editing
TALENs (Transcription Activator-Like Effector Nucleases	Protein – DNA binding	-High specificity and low off- target effects -Flexible for targeting any sequences	more complex design and assemble as compared to CRISPR
Synthetic Gene Drives	CRISPR based or other genetic elements promoting inheritance	-Power to control vector borne disease	Risk of unintended ecological consequences
CRISPR-Cas12 and Cas13	RNA guided DNA (cas2) or RNA (cas13) endonuclease	-High flexibility - Potential for use in diagnostics (e.g., SHERLOCK, DETECTR	Limited target range
Meganucleases (Homing Endonucleases)	DNA endonuclease with large recognition sites	High specificity due to large recognition sites	Hard to engineer for new target sites
RNA Editing (ADAR-based)	RNA editing by adenosine deaminase acting on RNA	-Allow reversible and transient modification at RNA level - Avoid permanent DNA modification	Lower efficiency as compared to DNA editing techniques
Prime Editing	Reverse transcriptase coupled with CRISPR- Cas9	- Capability to make a wide variety of precise edits, together with insertions, deletions, and all types of bases changes.	More complex than CRISPR- Cas9
Base Editing	Deaminase enzymes coupled with CRISPR-Cas9 or other nucleases	- Accurate single nucleotide variations without forming double-strand - Lesser risk of off-target effects compared to CRISPR	Limited to certain types of base changes (e.g., C to T, A to G)
ZFNs (Zinc Finger Nucleases)	Protein- DNA binding	-Appropriate for numerous cell types -Customizable to recognize specific DNA sequences. -High specificity	High cost Complexity in design potential off- target effects.

## 4 Future prospective

Multiple signaling pathways can trigger inflammatory responses in the body, and dysfunction of the immunoproteasome has recently emerged as an important contributor within this network. The clinical features seen in patients with inherited PSMB8 mutations highlight the essential role of the immunoproteasome in maintaining immune balance. However, many aspects of its clinical relevance remain poorly understood. Future research should focus on the following key areas.

I. Elucidating the biochemical and structural changes caused by PSMB8 mutations, particularly how these alterations interfere with the proper integration of PSMB8 within the immunoproteasome [80].
II. Developing more representative animal models that better mirror the inflammatory features and disease patterns observed in affected patients.
III. Identifying different genetic variants in immunoproteasome components and determining how these variations modulate inflammatory responses and proteasome function.
IV. Clarifying the specific factors and triggers that lead to increased IL-6 production in affected individuals [81]**.**
V. Understanding how changes in immunoproteasome activity influence the buildup of oxidized proteins, and how this accumulation contributes to the onset and progression of autoinflammatory conditions.
VI. Investigating the diverse ways in which the immunoproteasome shapes immune and inflammatory pathways, including its unexpected or contradictory roles. Notably, patients with PSMB8 mutations often display unique cytokine patterns: some have persistently high IL-6 levels, while others show increased IFN-γ. These differences likely stem from the distinct effects that specific mutations exert on immunoproteasome assembly and function. Establishing suitable animal models will help clarify which cytokines play a central role in driving inflammation in JASL [82]**.**
VII. Exploring how chronic joint inflammation is linked to the immune dysregulation seen in JASL [83]**.** Mapping the molecular pathways responsible for persistent inflammation will also help explain the development of fibrotic changes that arise due to immunoproteasome insufficiency [84]**.**
VIII. Finally, it remains an open and important question whether immunoproteasome defects also contribute to more widespread inflammatory diseases. Understanding how immunoproteasomes differ functionally from constitutive proteasomes and whether they uniquely regulate inflammatory suppression could reveal new therapeutic targets. If the immunoproteasome's role in adipocyte differentiation and inflammation is relevant beyond rare disorders, then controlling its activity may offer new strategies to reduce chronic inflammation or abnormal fat accumulation. Addressing these unresolved questions will deepen our understanding of immunoproteasome biology and may guide the development of new treatments for a broad range of inflammatory illnesses. Neutrophilic Dermatosis with Lipodystrophy and Elevated Temperature (CANDLE), STING-Associated Vasculopathy with Onset in Infancy (SAVI), Aicardi–Goutières syndrome (AGS), and certain monogenic forms of systemic lupus erythematosus (monoSLE) represent key examples of type I interferon–driven disorders. The monoSLE subgroup is typically linked to loss-of-function mutations in the DNA-degrading enzymes DNASE1 and DNASE1L3, which impair the clearance of extracellular or apoptotic DNA and trigger inappropriate interferon activation. In patients with CANDLE syndrome, skin biopsies commonly show dense infiltrates of neutrophils and atypical mononuclear cells, reflecting the underlying chronic inflammatory state characteristic of this condition [85].

## 5 Vaccinations

CANDLE syndrome is an uncommon autoinflammatory condition with recurring fever, skin lesions, and systemic inflammatory symptoms. Vaccination is not a therapeutic treatment for this disease. Because of their weakened immune systems and preexisting medical issues, people with CANDLE syndrome may have difficulties receiving the vaccination. To ensure the safety and efficacy of immunization in this population, careful evaluation of the vaccine types, timing, and potential dangers is necessary. People with CANDLE syndrome should receive specific vaccinations, such as those targeting pneumococcal disease, influenza, and other preventable illnesses, to help lower the risk of infections and accompanying problems [86]. For those with CANDLE syndrome, speaking with medical professionals who specialize in immunodeficiency disorders is essential in determining the right immunization regimen and timing. It takes careful observation and investigation to guarantee the best possible protection. Vaccinations are typically safe, but because of their underlying medical issues, people with CANDLE syndrome may be more prone to negative reactions. Minimizing the risks associated with vaccination in this population requires close monitoring for any possible adverse effects and quick medical intervention in cases of concern [87]. Adult CANDLE syndrome patients should receive vaccinations based on clinical judgment and evidence-based guidelines. Studies that specifically address vaccine effectiveness, immunogenicity, and safety in this population may be few due to the rarity of CANDLE syndrome. These recommendations, however, can be derived from general immunization guidelines and related situations for immunocompromised patients. The Task Force advises against administering live vaccinations to patients with CANDLE/PRAAS who are receiving therapy with JAKI or other immunosuppressive drugs, in accordance with the general EULAR guidance. When treatment is stopped, withdrawal symptoms may worsen. Treatment modifications for type I interferonopathies, including JAKI, are currently not included in the recommendations for other autoimmune and inflammatory rheumatic disorders. As of right now, there is no data to support any particular advice [88].

## 6 Conclusion

New understandings of genetics and pathophysiology of CANDLE syndrome have been made possible by the recent identification of the condition and the extensive investigation of cases that have been reported. Proteosome immunoproteasome malfunction caused by gene mutations causes CANDLE syndrome, an AID. Genetic verification is required. Although early treatment is preferred to avoid incapacitating symptoms, no medication has shown to be fully successful to date. Patients will be able to live longer if acute inflammatory episodes are prevented and treated. Patients with PRAAS can go years without receiving a diagnosis and present as complex clinical presentations. Accurate diagnosis is difficult due to the similarity of test results and clinical presentation between PRAAS subgroups. Finding efficient treatments for these diseases will likely be aided by additional research into their pathophysiology. In addition to clinical, cutaneous, histopathological, and laboratory data, a conclusive diagnosis is confirmed upon the discovery of a pertinent genetic mutation associated with a particular illness. Despite the rarity of these illnesses, patients may suffer greatly from skin symptoms, fever episodes, and end-organ damage, such as compromised renal function due to systemic amyloidosis. The JAK inhibitor treatment of the CANDLE syndrome patient described here has reliable results. While JAK 1/2 inhibitors have great short-term results, there are still questions about their long-term efficacy and potential for major side effects. The cost and accessibility of genetic testing, interferon signature assays, and JAKI medicines are currently some of the barriers that keep people with interferonopathies from receiving the best possible treatment. In addition, people with the autoinflammatory interferonopathies CANDLE are treated in various healthcare systems and reside in a variety of nations.

## CRediT authorship contribution statement

**Shivam Singh:** Writing – review & editing, Visualization, Supervision, Software, Project administration, Methodology, Investigation, Funding acquisition, Formal analysis, Conceptualization. **Ashish Kumar Sharma:** Visualization, Supervision, Project administration, Conceptualization.

## Declaration of competing interest

The authors declare that they have no known competing financial interests or personal relationships that could have appeared to influence the work reported in this manuscript. All revisions made were solely in response to the reviewer's scientific comments, and no external funding, commercial involvement, or conflicts of interest were associated with the preparation of this manuscript.

## Data Availability

The authors do not have permission to share data.
